# Supplementary material for: Widespread bacterial diversity within the bacteriome of fungi
Source: Commun Biol. 2021 Oct 7;4:1168. doi: 10.1038/s42003-021-02693-y (PMC8497576; doi:10.1038/s42003-021-02693-y)
Supplement: Supplementary file 2 — Supplementary Figures [file 42003_2021_2693_MOESM2_ESM.pdf]

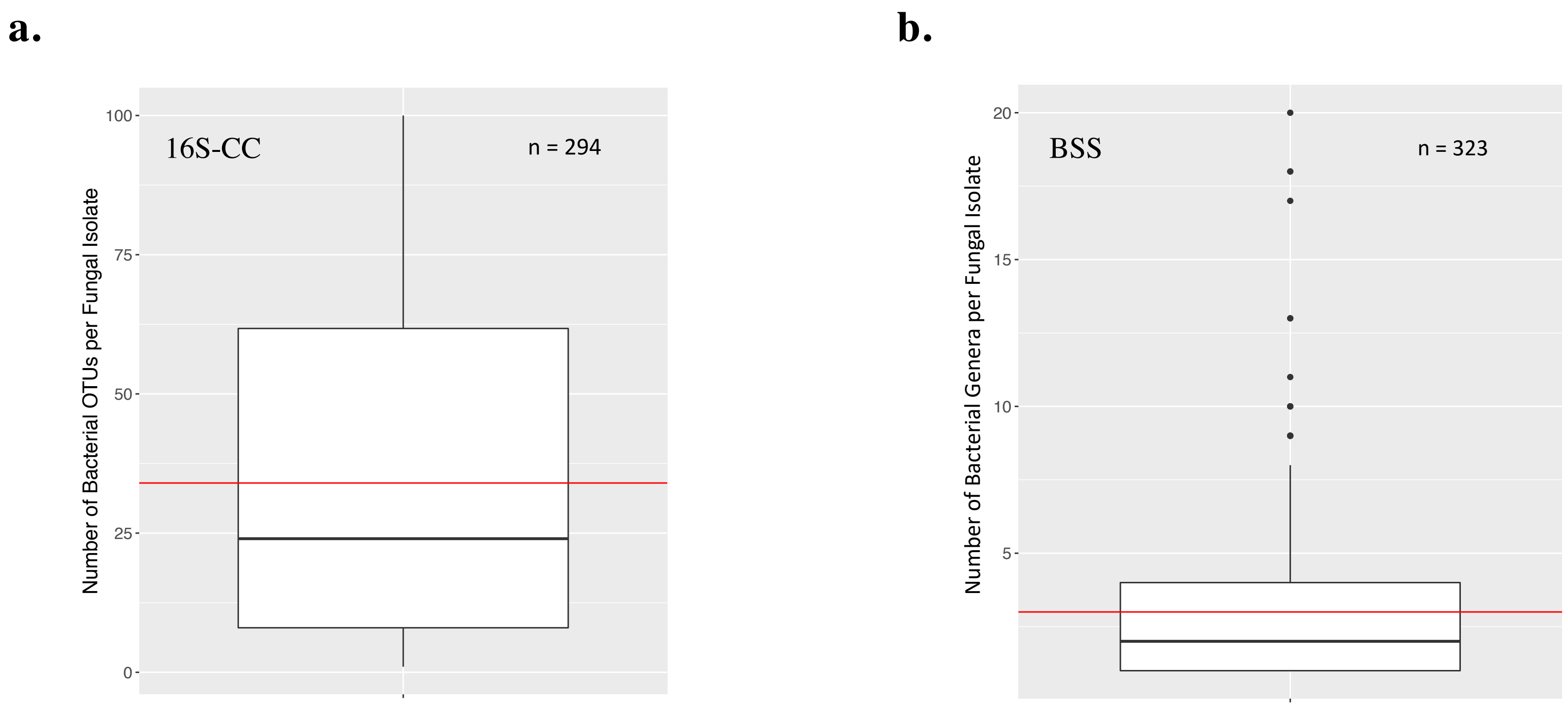

Supplementary Figure 1. Distribution of bacterial associates detected per fungal isolate. The number of detected **a)** bacterial OTUs per fungal isolate are shown for the 16S-CC screen, and the number of detected **b)** bacterial genera per fungal isolate are shown for the BSS screen. Mean values are indicated with a red line and the number of examined fungal isolates is shown in the upper right (n =).

Y-axis:
frequency (number of times a specific bacterial OTU is found within the fungal collection)

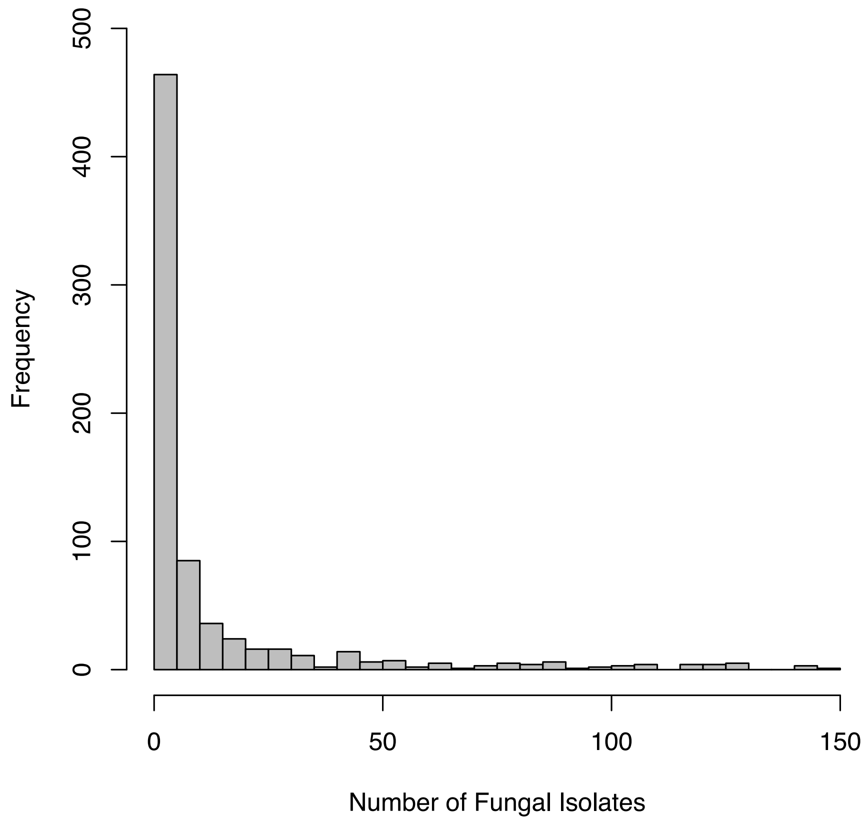

Supplementary Figure 2. Bacterial OTU detection frequency histogram. This histogram shows the number of times (y-axis) that bacterial OTUs were found in X (x-axis) fungal isolates in the 16S-CC screen. Among 294 fungal isolates, it was common to find specific bacterial OTUs in only 1-5 isolates, and very uncommon to find bacterial OTUs in 100-150 fungal isolates.

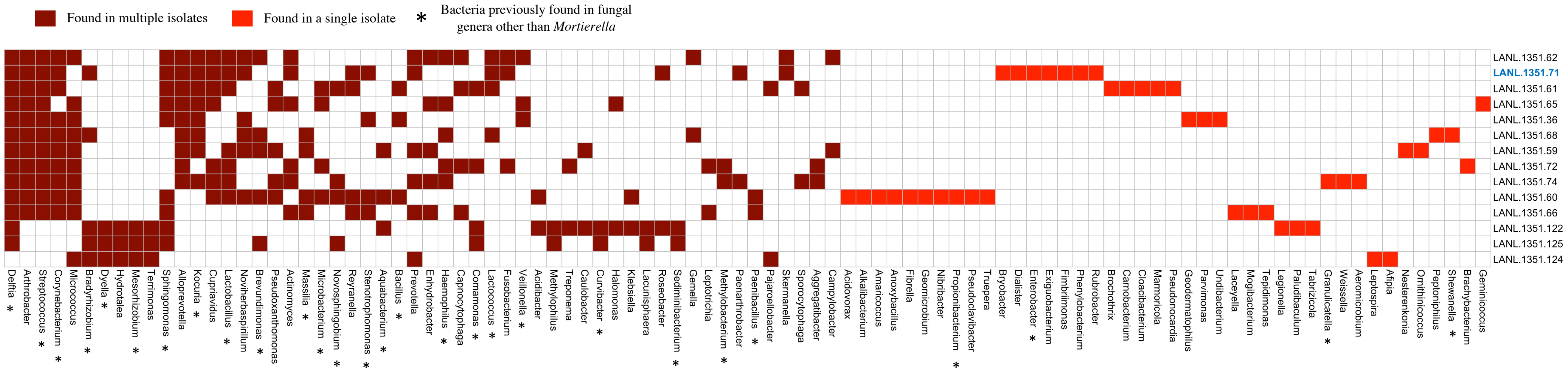

Supplementary Figure 3. Heatmap of bacterial genera detected among *Mortierella* isolates and a close relative *Podila* (labeled in blue) examined in the 16S-CC screen. Detected bacterial genera are represented in red, with darker red squares indicating bacterial genera found in multiple isolates (*Mortierella* and/or *Podila*) and lighter red indicating bacterial genera found in a single isolate (*Mortierella* or *Podila*). Bacterial genera described as fungal associates in previous studies are marked with an asterisk (*).

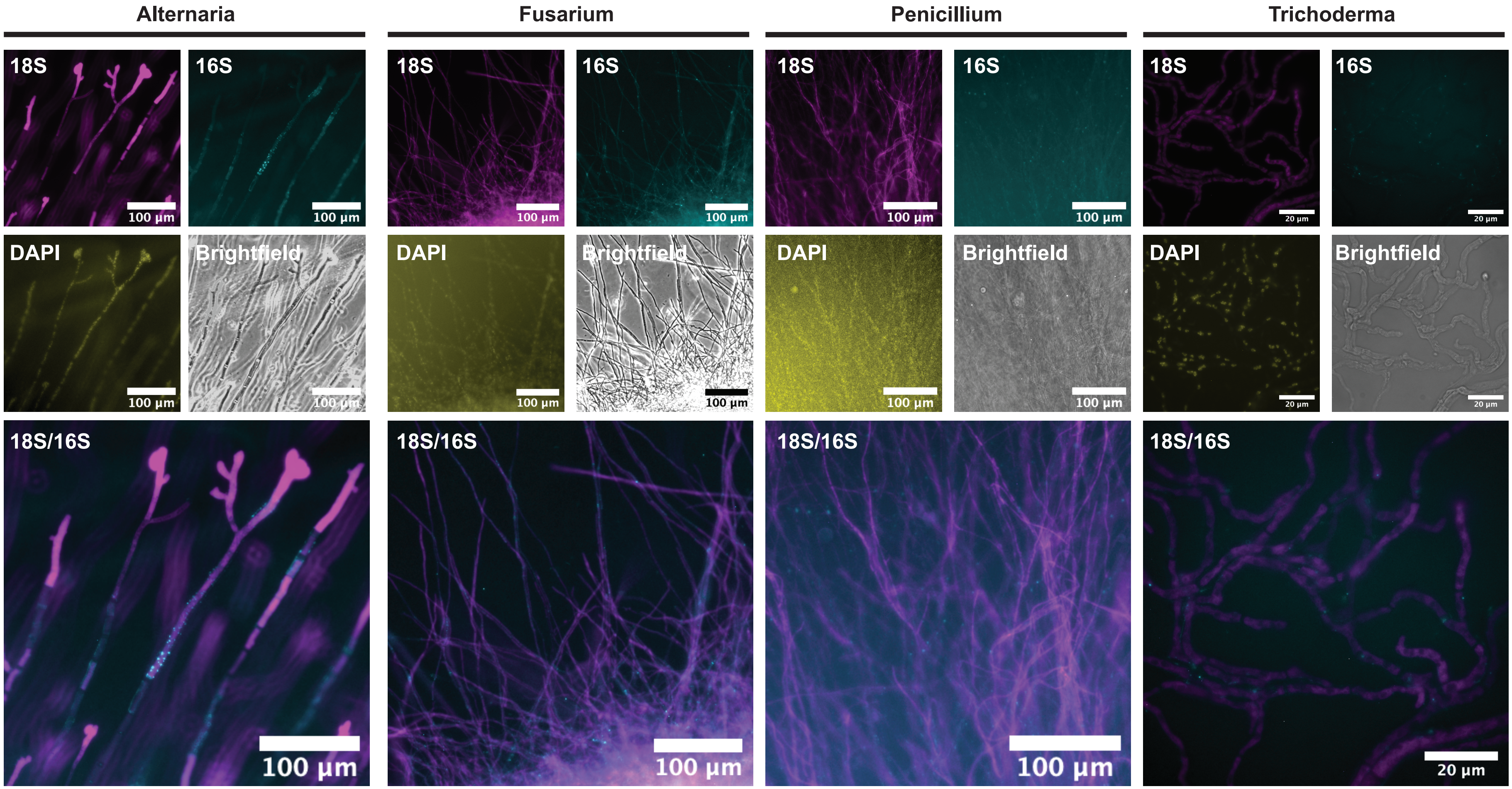

Supplementary Figure 4. High resolution-low magnification fluorescence *in situ* hybridization (FISH) images of select fungal isolates. These images provide a wider field of view of bacterial distribution across fungal isolates. For each fungal isolate, 16S rRNA was stained by HCR FISH (cyan) to detect bacterial associates, and a universal 18S rRNA probe (magenta) for the fungal rRNA. DAPI staining was used as a global DNA stain (yellow).

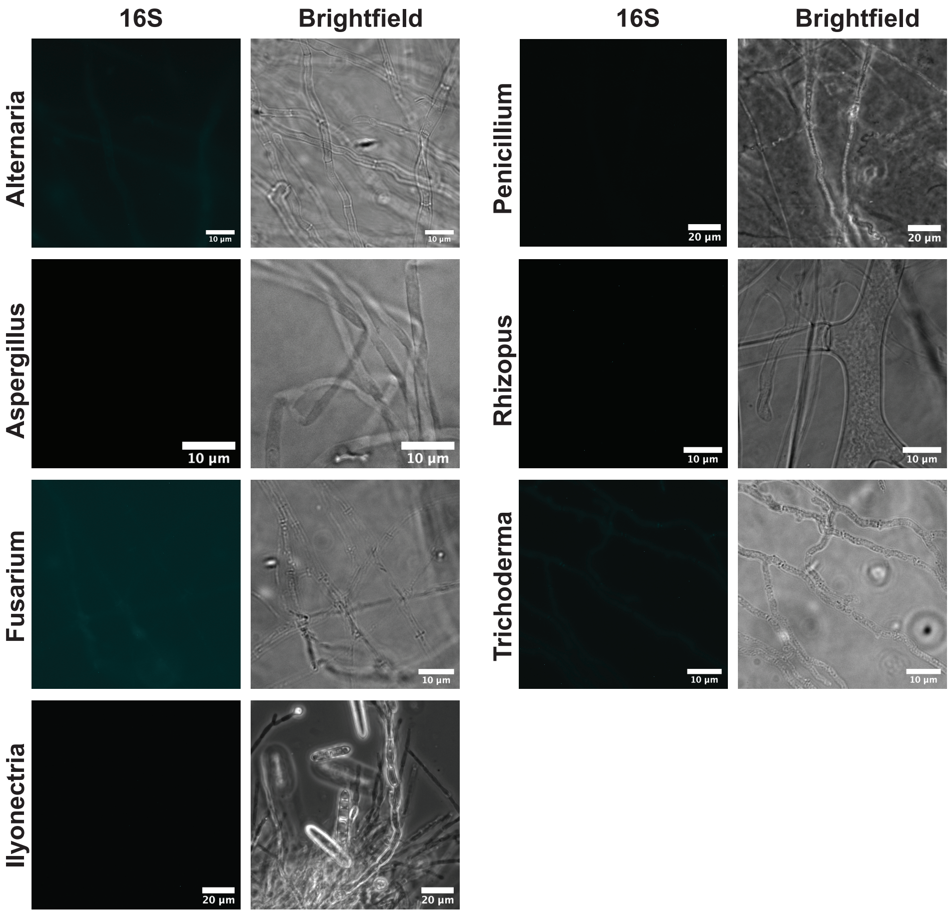

Supplementary Figure 5. Autofluorescence of fungal isolates. Autofluorescence of the fungal host was not observed at the same excitation wavelengths used by the 16S probes (cyan), indicating the absence of any background that may interfere with bacterial staining.

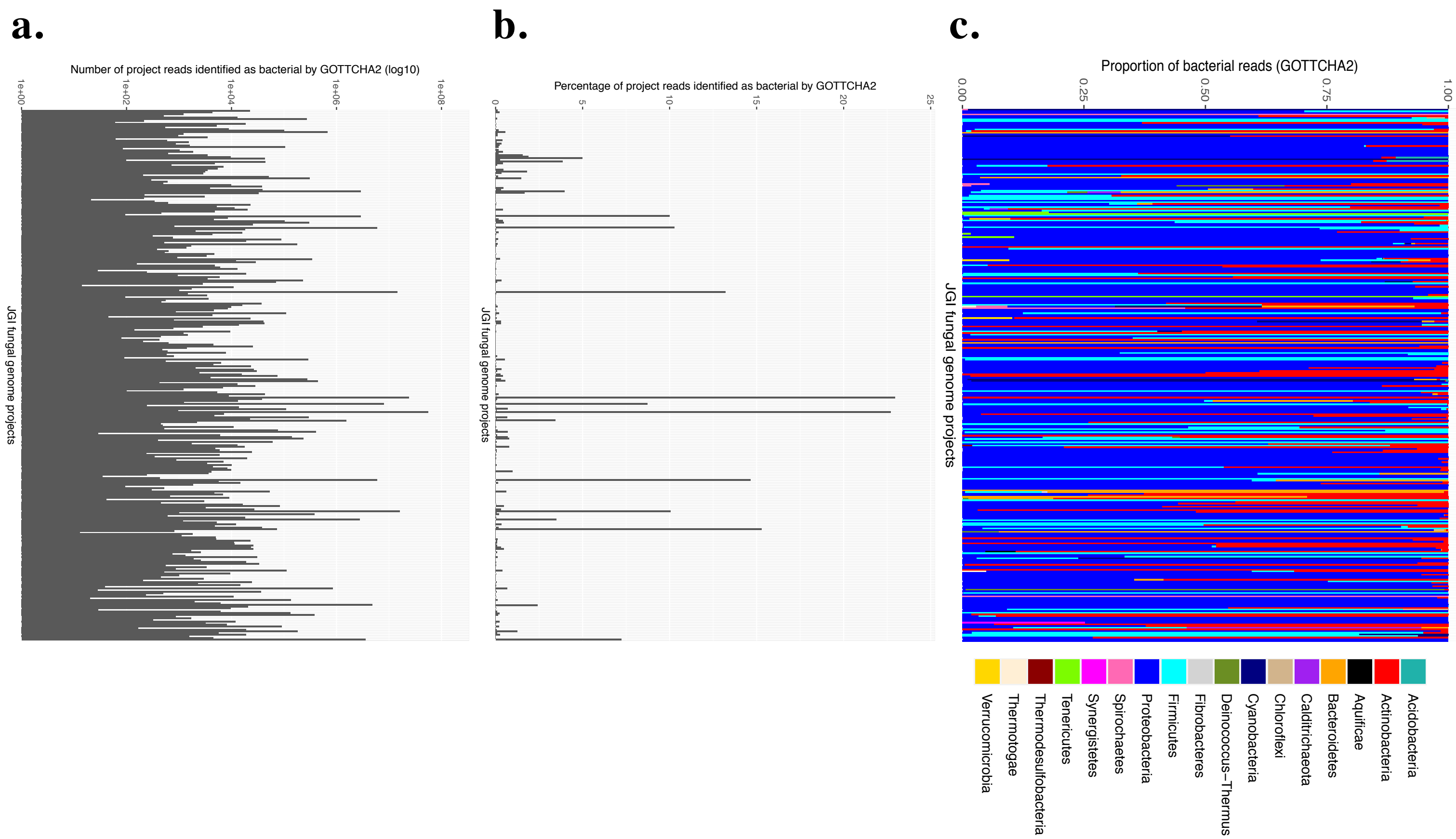

Supplementary Figure 6. Overview of bacterial data detected when screening public fungal genome sequencing projects with the bioinformatics signature screen. The **a)** number and **b)** percentage of sequencing reads identified as bacterial from each fungal sequencing project, as well as **c)** the taxonomic diversity of these data, represented proportionally at the phylum level are presented in this figure.

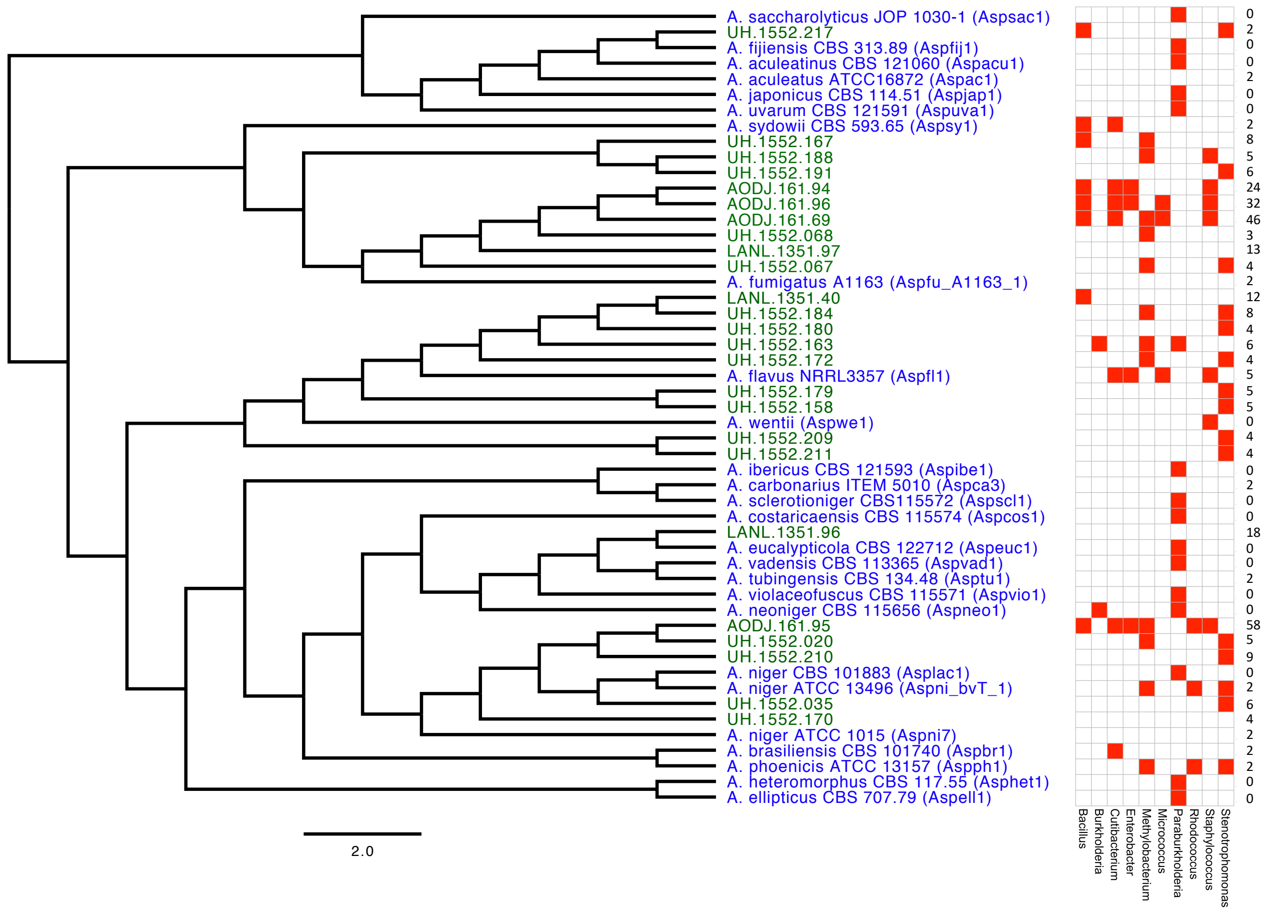

Supplementary Figure 7. Bacterial associates of *Aspergillus* isolates identified in the 16S-CC (green) and BSS (blue) screen. *Aspergillus* isolates are arranged by phylogenetic relatedness (ITS alignments) and red squares on the juxtaposed heatmap indicate which bacterial genera were detected in that isolate. The heatmap only shows bacterial genera identified in both screens and the numbers on the right indicate the number of additional bacterial genera (not shown in the heatmap) that were detected in each fungal isolate. Fungal isolates from the BSS screen include their JGI IDs (shown in parentheses).
