# Supplementary material for: Widespread bacterial diversity within the bacteriome of fungi
Source: Commun Biol. 2021 Oct 7;4:1168. doi: 10.1038/s42003-021-02693-y (PMC8497576; doi:10.1038/s42003-021-02693-y)
Supplement: Supplementary file 3 — Description of Additional Supplementary Files [file 42003_2021_2693_MOESM3_ESM.pdf]

Description of Additional Supplementary Files

File name: Supplementary Movie 1.
Description: Intra-hyphal localization confirmed by 3D projection of Lacunisphaera 16S staining by fluorescence in situ hybridization using a Lacunisphaera genus targeting probe set. The following key describes the staining in this figure: Lacunisphaera 16S probes (cyan), Aspergillus 18S probes (magenta), and DAPI probes (yellow).

File name: Supplementary Data 1.
Description: FASTA file of ITS sequences used to classify fungal isolates Page 7 of 17 examined in 16S-CC screen.

File name: Supplementary Data 2.
Description: Information on the origin (culture collection) and taxonomy for all fungal isolates analyzed in the 16SCC screen. The ITS sequences used for classification are included in the last column and are also available in FASTA format (Supplementary Data 1).

File name: Supplementary Data 3.
Description: Full list of all genus-level bacterial-fungal associations detected in either the 16S-CC screen, the BSS screen, or previous work. Associations described in previous work have links to the relevant publications.

File name: Supplementary Data 4.
Description: Summary heatmap of all putative bacterial associations observed with the 16S amplicon screen of diverse fungal isolates from multiple culture collections. Blue squares indicate when a bacterial taxon was detected in each isolate (presence/absence). Fungal isolates are presented on the y-axis, while bacterial OTUs resulting from the rollup of ASVs with identical taxonomic assignments at the genus level are presented on the x-axis.

File name: Supplementary Data 5.
Description: Genus-level bacterial-fungal associations detected in the 16S-CC screen with full taxonomic Page 8 of 17 information for both the fungal host and bacterial associate.

File name: Supplementary Data 6.
Description: ASV table generated by QIIME2 for the 16S amplicon screen of diverse fungal isolates from several culture collections. The identifier and relative abundance values for each ASV were determined during analysis with QIIME2.

File name: Supplementary Data 7.
Description: List of fungal genome datasets utilized in the BSS screen and their NCBI Sequence Read Archive derived metadata. Publications associated with this sequencing data are listed in the second column and the FTP address used to obtain data from each sequencing project is provided in the fourth column.

File name: Supplementary Data 8.
Description: Genus-level bacterial-fungal associations detected in the BSS screen with full taxonomic information for both the fungal host and bacterial associate.

File name: Supplementary Data 9.
Description: Genus and specieslevel GOTTCHA2 results and associated statistics for BSS screen of

fungal genome sequencing projects. The JGI project ID is listed together with identified bacteria at the genus level, NCBI bacterial taxonomic identifier, and GOTTCHA2 statistics for reads Page 9 of 17 mapped and genome coverage. A more detailed breakdown of the fields reported by GOTTCHA2 can be found at https://github.com/poeli/GOTTCHA2.

File name: Supplementary Data 10.
Description: FASTA file of representative sequences for each ASV identified by QIIME2 for the 16S-CC screen.

File name: Supplementary Data 11.
Description: Taxonomic assignments and confidence values for each ASV identified by QIIME2. The taxonomic assignments (second column) were determined by referencing the SILVA 16S rRNA sequence collection (SSU 138 Ref NR 99) and the confidence values in the third column (0.0 - 1.0) were also determined during analysis with QIIME2.

File name: Supplementary Data 12.
Description: Bacterial ASVs detected in the no-template (NTC) or DNA extraction control samples for each examined culture collection in the 16S-CC screen. Each control is labeled with both the type of control and which collection it belongs to. All ASVs represented in this table were identified in the original QIIME2 analysis (Supplementary Data 6 and Supplementary Data 11).

File name: Supplementary Data 13.
Description: Text file containing detailed information and Page 10 of 17 parameters for the in-silico design of taxonomically specific FISH probes.

File name: Supplementary Data 14.
Description: FASTA file containing sequences of taxonomically specific FISH probes.
