# Supplementary material for: Widespread bacterial diversity within the bacteriome of fungi
Source: Commun Biol. 2021 Oct 7;4:1168. doi: 10.1038/s42003-021-02693-y (PMC8497576; doi:10.1038/s42003-021-02693-y)
Supplement: Supplementary file 17 — Supplementary Data 13 [file 42003_2021_2693_MOESM17_ESM.docx]

Taxonomically specific FISH probe design parameters:

- Length: 18-22bp
- Tm: 42-47C (61-66C w/o formamide)
- GC: 40-60%
- Na+ conc: 390 mM
- Probe conc: 25 nM
- Formamide conc: 30%
  - Formamide lowers melting temperatures (Tm) of DNAs linearly by 2.4-2.9 degrees C/mole of formamide
  - [[Bio.SeqUtils.MeltingTemp.chem_correction](https://biopython.org/docs/1.75/api/Bio.SeqUtils.MeltingTemp.html#Bio.SeqUtils.MeltingTemp.chem_correction)]
    - Tm = Tm - factor(%formamide) (Default)
    - Tm + (0.453(f(GC)) - 2.88) x [formamide]

**Major workflows:**

- Based on the taxonomy assignment, the lineage level D_5 is used to separate ASV into 32 groups.
- We used OligoMiner [1] to design probes for each group, basically following these steps:
  - Masked repetitive sequences, homopolymeric runs and ambiguous bases from all sequences in the group.
  - Identified candidate probes with the provided criteria like the range of length and melting temperature.
  - Aligned candidate probes to all off target sequences including out-of-the-group (the other 31 groups) and eukaryotic rRNA sequences.
    - Eukaryotic rRNA sequences are downloaded and extracted from SILVA 138.1 ([ftp.arb-silva.de/current/Exports/](http://ftp.arb-silva.de/current/Exports/)) LSURef and SSURef.
  - The alignments were used to filter out non-specific probes with the recommended LDA model.
  - Further, we filtered out candidate probes with high abundance kmers and secondary structures.
- Perform another layer of specificity checking with Aspergillus genomes
  - A total of 41 Aspergillus (taxid: 5052) genome sequence are retrieved from NCBI using NCBI Datasets. Here is the full list:
    - GCF_000002655.1, GCF_000002715.2, GCF_000002855.3, GCF_000006275.2, GCF_000149205.2, GCF_000149615.1, GCF_000149645.2, GCF_000184455.2, GCF_001204775.2, GCF_001792695.1, GCF_001890805.1, GCF_001890905.1, GCF_002237265.1, GCF_002847045.1, GCF_002847465.1, GCF_002847485.1, GCF_002849105.1, GCF_003184525.1, GCF_003184535.1, GCF_003184545.1, GCF_003184585.1, GCF_003184595.1, GCF_003184625.1, GCF_003184695.1, GCF_003184745.1, GCF_003184755.1, GCF_003184765.1, GCF_003184785.1, GCF_003184835.1, GCF_003184845.1, GCF_003184865.1, GCF_003184925.1, GCF_003344945.1, GCF_003369625.1, GCF_003426965.1, GCF_009176365.1, GCF_009193445.1, GCF_009193585.1, GCF_009193645.1, GCF_010724455.1, GCF_013340325.1
  - Predicted non-specific binding to Aspergillus genomes are calculated using ThermonucleotideBLAST [2] with melting temperature calculated using standard nearest-neighbor thermodynamic parameters at 55C.
- Perform multiplexible checking for for qualified probes using ThermonucleotideBLAST.

Probe sequences used for *Lacunisphaera* targeting:

5’-3’

GAAGACCTTCATCCCTCACG

CCGGTGACAGAGGTTTACAAT

CGGGTTAAATCAGGCTTTGAAC

GAGTTAGCCACTGCTTCCTC

TCCGAATAACGCTTGCAGTC

TCACACCTTACACACACGG

TTTAAGGCGCAGTTCCGGG

CCCTCTCCAATACTCTAGCCA

TGCTACACCGTGAATTCCAC

GTTCCTCACGATATCTACGCAT

TGTCCAGGTAGTCGCCTTCG
