# Supplementary material for: Widespread bacterial diversity within the bacteriome of fungi
Source: Commun Biol. 2021 Oct 7;4:1168. doi: 10.1038/s42003-021-02693-y (PMC8497576; doi:10.1038/s42003-021-02693-y)
Supplement: Supplementary file 19 — Reporting Summary [file 42003_2021_2693_MOESM19_ESM.pdf]

nature research

Corresponding author(s): Patrick Chain

Last updated by author(s): Aug 27, 2021

Reporting Summary

Nature Research wishes to improve the reproducibility of the work that we publish. This form provides structure for consistency and transparency in reporting. For further information on Nature Research policies, see our Editorial Policies and the Editorial Policy Checklist.

Statistics

For all statistical analyses, confirm that the following items are present in the figure legend, table legend, main text, or Methods section.

n/a	Confirmed	
☐	☒	The exact sample size (*n*) for each experimental group/condition, given as a discrete number and unit of measurement
☐	☒	A statement on whether measurements were taken from distinct samples or whether the same sample was measured repeatedly
☒	☐	The statistical test(s) used AND whether they are one- or two-sided<br>*Only common tests should be described solely by name; describe more complex techniques in the Methods section.*
☒	☐	A description of all covariates tested
☒	☐	A description of any assumptions or corrections, such as tests of normality and adjustment for multiple comparisons
☐	☒	A full description of the statistical parameters including central tendency (e.g. means) or other basic estimates (e.g. regression coefficient) AND variation (e.g. standard deviation) or associated estimates of uncertainty (e.g. confidence intervals)
☒	☐	For null hypothesis testing, the test statistic (e.g. *F*, *t*, *r*) with confidence intervals, effect sizes, degrees of freedom and *P* value noted<br>*Give P values as exact values whenever suitable.*
☒	☐	For Bayesian analysis, information on the choice of priors and Markov chain Monte Carlo settings
☒	☐	For hierarchical and complex designs, identification of the appropriate level for tests and full reporting of outcomes
☒	☐	Estimates of effect sizes (e.g. Cohen's *d*, Pearson's *r*), indicating how they were calculated

Our web collection on statistics for biologists contains articles on many of the points above.

Software and code

Policy information about availability of computer code

Data collection	No software was used.
Data analysis	QIIME 2 release v2021.4.0; jgi-query script v1.0.1 (https://github.com/glarue/jgi-query); SRAdb release 3.13 (https://bioconductor.org/packages/release/bioc/html/SRAdb.html); FaQCs release 2.10 (https://github.com/LANL-Bioinformatics/FaQCs); EDGE-UI v2.4.0 (https://edgebioinformatics.org); BWA release 0.7.17 (https://github.com/lh3/bwa); GOTTCHA2 v2.1.7 (https://github.com/poeli/GOTTCHA2); PhaME v1.0.4 (https://github.com/LANL-Bioinformatics/PhaME), RAxML v8.2.12; OligoMiner v1.0 (https://github.com/beliveau-lab/OligoMiner); thermonucleotideBLAST v2.2 (https://github.com/jgans/thermonucleotideBLAST)

For manuscripts utilizing custom algorithms or software that are central to the research but not yet described in published literature, software must be made available to editors and reviewers. We strongly encourage code deposition in a community repository (e.g. GitHub). See the Nature Research guidelines for submitting code & software for further information.

Data

Policy information about availability of data

All manuscripts must include a data availability statement. This statement should provide the following information, where applicable:

- Accession codes, unique identifiers, or web links for publicly available datasets
- A list of figures that have associated raw data
- A description of any restrictions on data availability

The ITS sequences used for classification of the fungal isolates have been deposited at NCBI GenBank and the unprocessed 16S amplicon sequencing data obtained from these isolates have been deposited in the NCBI SRA database (BioProject accession number: PRJNA738181). All other source data is contained within the supplemental material, which are available through figshare: https://doi.org/10.6084/m9.figshare.c.5582283.v1.

Field-specific reporting

Please select the one below that is the best fit for your research. If you are not sure, read the appropriate sections before making your selection.

- [x] Life sciences
- [ ] Behavioural & social sciences
- [ ] Ecological, evolutionary & environmental sciences

For a reference copy of the document with all sections, see nature.com/documents/nr-reporting-summary-flat.pdf

Life sciences study design

All studies must disclose on these points even when the disclosure is negative.

Sample size	702 fungal isolates were examined in this work. To gain a more comprehensive view of the diversity of bacterial-fungal associations, we employed two complementary approaches to identify signals of potential bacterial associates among a phylogenetically broad range of fungi. We analyzed 16S ribosomal RNA (rRNA) gene amplicon sequences obtained from total DNA extractions of distinct fungal isolates belonging to four different fungal culture collections from diverse environments within North America, South America and Europe (hereafter referred to as 16S-CC screen). We also searched for bacterial-specific sequences (hereafter referred to as BSS screen) within publicly available fungal genome sequencing projects from the Joint Genome Institute (JGI) Mycocosm portal,18 deliberately sampling the widest possible range of fungal phylogenetic diversity. This work provides a considerably more comprehensive exploration of the fungal bacteriome by examining over 700 fungal isolates, including 366 fungal genera (nearly ten times the amount in all previous examinations) and multiple representatives from nearly every fungal phylum.
Data exclusions	All bacterial genera represented by ASVs identified in the no-template (NTC) or DNA extraction control samples (Supplementary Table 9) were excluded from further analysis within the corresponding culture collection (e.g. if a single Bacillus ASV was found in any NTC sample, all Bacillus ASVs were excluded from that collection's analysis). Separate controls were used for each culture collection to try and minimize generalizing contaminants across each collection, as there was not a case where a contaminant ASV or taxa was found in all control samples (likely reflecting the fact that DNA extractions, PCR amplification and sequencing were performed in multiple laboratories).<br><br>Non-bacterial (mitochondria, chloroplast and other) ASVs were identified and were excluded from analyses, given our sole interest in bacterial associates.<br><br>A total of 22 taxa identified by SILVA were absent from the NCBI taxonomy database when comparing taxonomic classifications and were therefore excluded.
Replication	No biological replicates were utilized in this study and no statistical analyses are reported.
Randomization	This is not relevant to our study as no statistical analyses are reported.
Blinding	Blinding was not relevant to our study as we were profiling fungal bacteriomes.

Reporting for specific materials, systems and methods

We require information from authors about some types of materials, experimental systems and methods used in many studies. Here, indicate whether each material, system or method listed is relevant to your study. If you are not sure if a list item applies to your research, read the appropriate section before selecting a response.

Materials & experimental systems

n/a	Involved in the study	
x		Antibodies
x		Eukaryotic cell lines
x		Palaeontology and archaeology
x		Animals and other organisms
x		Human research participants
x		Clinical data
x		Dual use research of concern

Methods

n/a	Involved in the study	
x		ChIP-seq
x		Flow cytometry
x		MRI-based neuroimaging
